# Histological Characterization of the Tumorigenic “Peri-Necrotic Niche” Harboring Quiescent Stem-Like Tumor Cells in Glioblastoma

**DOI:** 10.1371/journal.pone.0147366

**Published:** 2016-01-22

**Authors:** Aya Ishii, Tokuhiro Kimura, Hirokazu Sadahiro, Hiroo Kawano, Keiyo Takubo, Michiyasu Suzuki, Eiji Ikeda

**Affiliations:** 1 Department of Pathology, Yamaguchi University Graduate School of Medicine, Ube, Yamaguchi, Japan; 2 Department of Neurosurgery, Yamaguchi University Graduate School of Medicine, Ube, Yamaguchi, Japan; 3 Department of Basic Laboratory Sciences, Yamaguchi University Graduate School of Medicine, Ube, Yamaguchi, Japan; 4 Research Institute National Center for Global Health and Medicine, Shinjuku, Tokyo, Japan; University of Alabama at Birmingham, UNITED STATES

## Abstract

**Background:**

Characterization of the niches for stem-like tumor cells is important to understand and control the behavior of glioblastomas. Cell-cycle quiescence might be a common mechanism underlying the long-term maintenance of stem-cell function in normal and neoplastic stem cells, and our previous study demonstrated that quiescence induced by hypoxia-inducible factor (HIF)-1α is associated with a high long-term repopulation capacity of hematopoietic stem cells. Based on this, we examined human astrocytoma tissues for HIF-1α-regulated quiescent stem-like tumor cells as a candidate for long-term tumorigenic cells and characterized their niche histologically.

**Methods:**

Multi-color immunohistochemistry was used to visualize HIF-1α-expressing (HIF-1α^+^) quiescent stem-like tumor cells and their niche in astrocytoma (WHO grade II–IV) tissues. This niche was modeled using spheroids of cultured glioblastoma cells and its contribution to tumorigenicity was evaluated by sphere formation assay.

**Results:**

A small subpopulation of HIF-1α^+^ quiescent stem-like tumor cells was found in glioblastomas but not in lower-grade astrocytomas. These cells were concentrated in the zone between large ischemic necroses and blood vessels and were closer to the necrotic tissues than to the blood vessels, which suggested that a moderately hypoxic microenvironment is their niche. We successfully modeled this niche containing cells of HIF-1α^+^ quiescent stem-like phenotype by incubating glioblastoma cell spheroids under an appropriately hypoxic condition, and the emergence of HIF-1α^+^ quiescent stem-like cells was shown to be associated with an enhanced sphere-forming activity.

**Conclusions:**

These data suggest that the “peri-necrotic niche” harboring HIF-1α^+^ quiescent stem-like cells confers a higher tumorigenic potential on glioblastoma cells and therefore may be a therapeutic target to control the behavior of glioblastomas.

## Introduction

Astrocytic tumors are the most common tumors arising in the central nervous system. According to the WHO classification system [1], infiltrating astrocytic tumors are classified into diffuse astrocytoma (grade II), anaplastic astrocytoma (grade III), and glioblastoma (grade IV). This grading system reflects the biological behavior of astrocytic tumors, with glioblastoma having the poorest prognosis among them. Despite advances in surgery, radiotherapy, and chemotherapy, the prognosis of glioblastoma remains unfavorable, with a reported 2-year survival rate of only 3.3% [2]. Therefore, to eradicate the tumor cells, a better understanding of the pathophysiology of glioblastoma, including the determination of real therapeutic targets, is urgently needed.

Cancer stem cells (CSCs) are a subpopulation of tumor cells with stem-like properties which have self-renewal capacity, can give rise to heterogeneous cells that comprise a tumor [3], and are thought to be responsible for the tumorigenesis, maintenance, and recurrence of the tumor. In gliomas, glioma stem cells (GSCs) were described [4, 5] and have been analyzed based on their expression of “stem-cell markers” such as CD133 [5], CD15 [6], and CD44 [7] or their phenotype of “side population” as evaluated by flow cytometry [8], but there has been no consensus on the marker phenotypes of GSCs [3, 9]. Therefore, it is possible that there has been no successful visualization of GSCs that are the real culprits determining the poor prognosis of glioblastoma. From the pathophysiological point of view, one of the cardinal characteristics of glioblastoma is intratumoral heterogeneity in microenvironments [10]. For example, a glioblastoma tissue is composed of various regions from well-vascularized areas to severely hypoxic necrotic areas. This microenvironmental heterogeneity has various effects on the properties of tumor cells and consequently influences the pathophysiology of the tumor. It is assumed that GSCs are not distributed randomly in the tumor tissue, but are localized in a specialized niche that confers high tumorigenic potential on tumor cells [3, 11]. Although recent reports have described some evidence for the roles of a perivascular niche [12] and a hypoxic niche [13] in the pathophysiology of glioblastoma, precise *in situ* visualization and characterization of the niche that harbors GSCs have not been fully established.

Accumulating evidence has highlighted the importance of being in a quiescent state for stem cells of normal adult tissues to maintain their high capacity for tissue repopulation, such as that occurring after bone marrow transplantation [14, 15]. Similarly, as for CSCs, a quiescent subpopulation of tumor cells was reported to be responsible for tumor-propagating capacity in leukemia [16], pancreatic cancer [17], breast cancer [18], melanoma [19], and glioblastoma [20]. It was also reported, using a mouse model that spontaneously develops a malignant glioma, that a subpopulation of Ki67-negative quiescent tumor cells is responsible for tumor regrowth after chemotherapy [21]. These lines of evidence compelled us to examine histological sections for stem-like tumor cells in a quiescent state, in order to determine the niche harboring the most important cells regulating the behavior of glioblastoma. Increasing evidence has indicated that hypoxia-inducible factor (HIF)-1α, which is a subunit of HIF-1 and a master regulator of the cellular response to tissue hypoxia [22], is involved in the promotion and maintenance of the stem-cell properties of glioma cells [23–25]. In addition, we recently demonstrated that the expression of HIF-1α protein at an appropriate level is essential for hematopoietic stem cells (HSCs) to maintain their quiescence and long-term repopulation capacity [26]. Based on these findings, we hypothesized in this study that stem-like glioma cells showing HIF-1α-regulated quiescence are a strong candidate for long-term tumorigenic cells, and we tried to visualize these cells and their niches histologically in clinical tissue samples of astrocytic tumors.

Here, we demonstrated by multi-color immunohistochemical analysis of tissue samples of glioblastoma that HIF-1α-positive (HIF-1α^+^) quiescent stem-like tumor cells are characteristically found in the zone between large ischemic necroses and blood vessels, and are closer to the necrotic areas than to the blood vessels. By culturing glioblastoma cell spheroids under hypoxic conditions, we showed that HIF-1α^+^ quiescent stem-like cells emerge in a hypoxia-dependent manner and are associated with an enhanced sphere-forming activity. With these findings, we advocate a new candidate for the GSC niche, the “peri-necrotic niche”, which induces a higher tumorigenic potential and consequently regulates the behavior of glioblastomas.

## Materials and Methods

### Clinical tissue samples

We analyzed 40 cases of infiltrating astrocytic tumors that had been operated at Yamaguchi University Hospital. These cases consisted of 10 diffuse astrocytomas (grade II) (median [range] of age, 55 [20–70] years; male:female ratio, 7:3), 9 anaplastic astrocytomas (grade III) (49 [17–78] years; 7:2), and 21 glioblastomas (grade IV) (67 [48–81] years; 15:6). In all of these cases, the histological diagnosis was reviewed according to the latest WHO Classification [1]. Neutral buffered formalin-fixed paraffin-embedded sections of tumor tissues were prepared for immunohistochemical analyses. This study was approved by the Institutional Review Board of Yamaguchi University Hospital. Written informed consent was obtained from all of the individual participants included in the study.

### Indices of cellular stemness and quiescence

The proteins SOX2 and NANOG were chosen as markers of the stemness of tumor cells, since they are well-known as core transcription factors involved in the self-renewal and pluripotency of embryonic stem cells [27] and were also reported to play critical roles in the tumorigenesis of glioblastoma cells [28, 29]. As an index of cell-cycle quiescence, we used suppressed phosphorylation at the serine (Ser) 2 residue of the C-terminal domain (CTD) of RNA polymerase II. Phosphorylation at the Ser2 residue of the CTD of RNA polymerase II is abbreviated to RNApII-S2P in the present study. The CTD of RNA polymerase II contains heptapeptide repeats of the consensus sequence Tyr1-Ser2-Pro3-Thr4-Ser5-Pro6-Ser7 [30]. Phosphorylation at Ser5 (RNApII-S5P) is required for the initiation of transcription, while RNApII-S2P triggers the productive elongation phase of transcription as well as mRNA processing [30]. A recent report [31] showed that the suppression of RNApII-S2P (RNApII-S2P^-/low^) was observed in quiescent stem cells in various normal tissues and suggested that this can be a marker to discriminate stem cells in a quiescent state from proliferating, terminally differentiated, and senescent cells [14, 31]. Positivity for RNApII-S5P was used as an index for the viability of RNApII-S2P^-/low^ cells.

### Single-color immunohistochemistry

For primary antibodies other than anti-carbonic anhydrase IX (CA IX) antibody, deparaffinized sections were pretreated for antigen retrieval by boiling in antigen retrieval solution, pH 9 (Nichirei Biosciences, Tokyo, Japan). No antigen-retrieval treatment was performed for anti-CA IX antibody. Endogenous peroxidase activity was blocked with peroxidase-blocking solution (Dako, Glostrup, Denmark). Sections were incubated with mouse monoclonal antibody against HIF-1α (1:20; clone 54/HIF-1α; BD Biosciences, Franklin Lakes, NJ, USA), mouse monoclonal antibody against HIF-2α (1:400; clone 190b; Millipore, Billerica, MA, USA), goat polyclonal antibody against SOX2 (1:100; Santa Cruz Biotechnology, Santa Cruz, CA, USA), goat polyclonal antibody against NANOG (1:250; Novus Biologicals, Littleton, CO, USA), phosphorylation-specific rabbit polyclonal antibody to detect RNApII-S2P (1:6000; Abcam, Cambridge, UK), rabbit monoclonal antibody against Ki-67 (1:1000; clone EPR3610; Epitomics, Burlingame, CA, USA), or rabbit polyclonal antibody against CA IX (1:200; Novus Biologicals). After the reaction with horseradish peroxidase (HRP)-conjugated secondary antibodies (EnVision+, Dako, for mouse and rabbit primary antibodies; anti-goat immunoglobulins/HRP, Dako, for goat primary antibodies), color was developed with 3,3′-diaminobenzidine (DAB) and sections were counterstained with hematoxylin. Tissue sections of human testicular seminoma and human renal cell carcinoma were used as positive controls for immunostaining for NANOG and CA IX, respectively.

### Chromogenic multi-color immunohistochemistry

For triple immunostaining for SOX2/HIF-1α/RNApII-S2P or NANOG/HIF-1α/RNApII-S2P in glioma tissues or spheroids, sections were pretreated for antigen retrieval and endogenous peroxidase blocking, and then incubated with anti-SOX2 or anti-NANOG antibody. After the reaction with alkaline phosphatase (AP)-conjugated donkey anti-goat IgG (H+L) antibody (Jackson ImmunoResearch Laboratories, West Grove, PA, USA), color was developed with Vulcan Fast Red (Biocare Medical, Concord, CA, USA). The sections were treated with Denaturing Solution (Biocare Medical) to ensure that the second staining did not cross-react with the first staining. After the sections were incubated successively with anti-HIF-1α antibody and phosphorylation-specific antibody to detect RNApII-S2P, MACH 2 Double Stain 1 (a secondary antibody cocktail containing AP-conjugated goat anti-mouse IgG and HRP-conjugated goat anti-rabbit IgG antibodies; Biocare Medical) was applied. Color was developed with Perma Blue/AP (Diagnostic BioSystems, Pleasanton, CA, USA) for HIF-1α and DAB for RNApII-S2P.

For triple immunostaining for estrogen receptor (ER), progesterone receptor (PgR), and Ki-67 in breast cancer tissue, the sections were pretreated as described above and incubated with mouse anti-ERα monoclonal antibody (1:80; clone 1D5; Dako). After the reaction with AP-conjugated donkey anti-mouse IgG (H+L) antibody (Jackson ImmunoResearch Laboratories), color was developed with Vulcan Fast Red. The sections were treated with Denaturing Solution and then incubated successively with mouse anti-PgR monoclonal antibody (1:100; clone PgR 636; Dako) and rabbit anti-Ki-67 monoclonal antibody. Subsequently, MACH 2 Double Stain 1 was applied, and color was developed with Perma Blue/AP for PgR and DAB for Ki-67.

The staining pattern of each antigen in multi-color immunohistochemistry was validated by comparing it with the staining pattern of the same antigen in single-color immunohistochemistry.

### Immunofluorescent staining

Sections were pretreated for antigen retrieval and then incubated with primary antibodies. Secondary antibodies and fluorescent dyes used for multi-color immunofluorescent staining were as follows: Alexa Fluor 546- or Alexa Fluor 633-conjugated donkey anti-goat IgG antibody (Molecular Probes, Eugene, OR, USA) for SOX2 or NANOG; biotinylated donkey anti-mouse IgG antibody and Alexa Fluor 488-conjugated streptavidin (Molecular Probes) for HIF-1α; Alexa Fluor 635- or Alexa Fluor 405-conjugated goat anti-rabbit IgG antibody (Molecular Probes) for RNApII-S2P. Phosphorylation-specific rabbit polyclonal antibody to detect RNApII-S5P (1:1000; Abcam) was directly labeled using Zenon Alexa Fluor 546 rabbit IgG labeling kit (Molecular Probes). For the staining of nuclei, 4′,6-diamidino-2-phenylindole (DAPI) was used. Stained sections were observed and analyzed with a confocal microscope, LSM510META (Carl Zeiss Microscopy, Jena, Germany), and differential interference contrast images were obtained. Digital images were processed using Zeiss LSM Image Browser software (Carl Zeiss Microscopy). In order to validate the results of chromogenic multi-color immunohistochemistry, 3 representative cases of glioblastomas were examined by multi-color immunofluorescent staining. In each case, 3–20 microscopic fields, each of which contained 100–300 cells, were analyzed.

### Cell culture

The human glioblastoma cell line T98G was obtained from American Type Culture Collection (Manassas, VA, USA). Cells were grown in minimum essential medium (Gibco, Life Technologies, Carlsbad, CA, USA) supplemented with 10% (v/v) fetal bovine serum (FBS; HyClone, South Logan, UT, USA). To generate multicellular spheroids, cells were seeded in a 60-mm culture dish coated with poly(2-hydroxyethyl methacrylate) (pHEMA; Sigma-Aldrich, St. Louis, MO, USA) at a density of 1 × 10^6^ cells/dish, and grown in serum-free Dulbecco’s modified Eagle’s medium/F-12 (Gibco, Life Technologies) supplemented with B-27 supplement (Gibco, Life Technologies), 20 ng/ml epidermal growth factor (Peprotech, Rocky Hill, NJ, USA), and 20 ng/ml basic fibroblast growth factor (Peprotech) [32] (stem cell medium). After the cells were cultured for 3 days, spheroids of 400–800 μm in diameter were formed, and then they were further incubated for 9 or 24 h under normoxic (5% CO_2_ and 95% atmospheric air) or hypoxic (5% CO_2_ and 5% O_2_ balanced with N_2_) conditions. A multi-gas incubator with an O_2_ control system (APM-50DR, ASTEC, Fukuoka, Japan) was used to generate the hypoxic culture conditions.

### Sphere formation assay

Sphere formation assay by limiting dilution analysis was performed as described previously [33, 34]. The spheroids were dissociated using 0.25% (w/v) trypsin-ethylenediaminetetraacetic acid (EDTA) solution (Sigma-Aldrich) and mechanically triturated using a 25G needle and syringe until a single cell suspension was achieved. After counting viable cell number by trypan blue exclusion, cells were plated by 2-fold serial dilution, resulting in a range from 1024 to 0.5 cells/well on a pHEMA-coated 96-well plate containing the stem cell medium in octuplicate for each cell density. After culture for 7 days under the normoxic condition, the presence or absence of spheres measuring more than 50 μm in diameter was recorded in each well. Sphere-forming efficiency was estimated as described previously [33].

### Statistical analysis

Mann-Whitney *U* test was used to compare the data from grade II, III, and IV tissue samples. Simple linear regression and Pearson’s correlation coefficient were used for the analysis of the relationships between the different parameters recorded in this study. For the experiments using cultured cells, statistical significance was determined by Student’s *t* test.

## Results

### HIF-1α^+^ quiescent stem-like tumor cells exist as a small subpopulation of human glioblastoma cells

**Fig 1:**
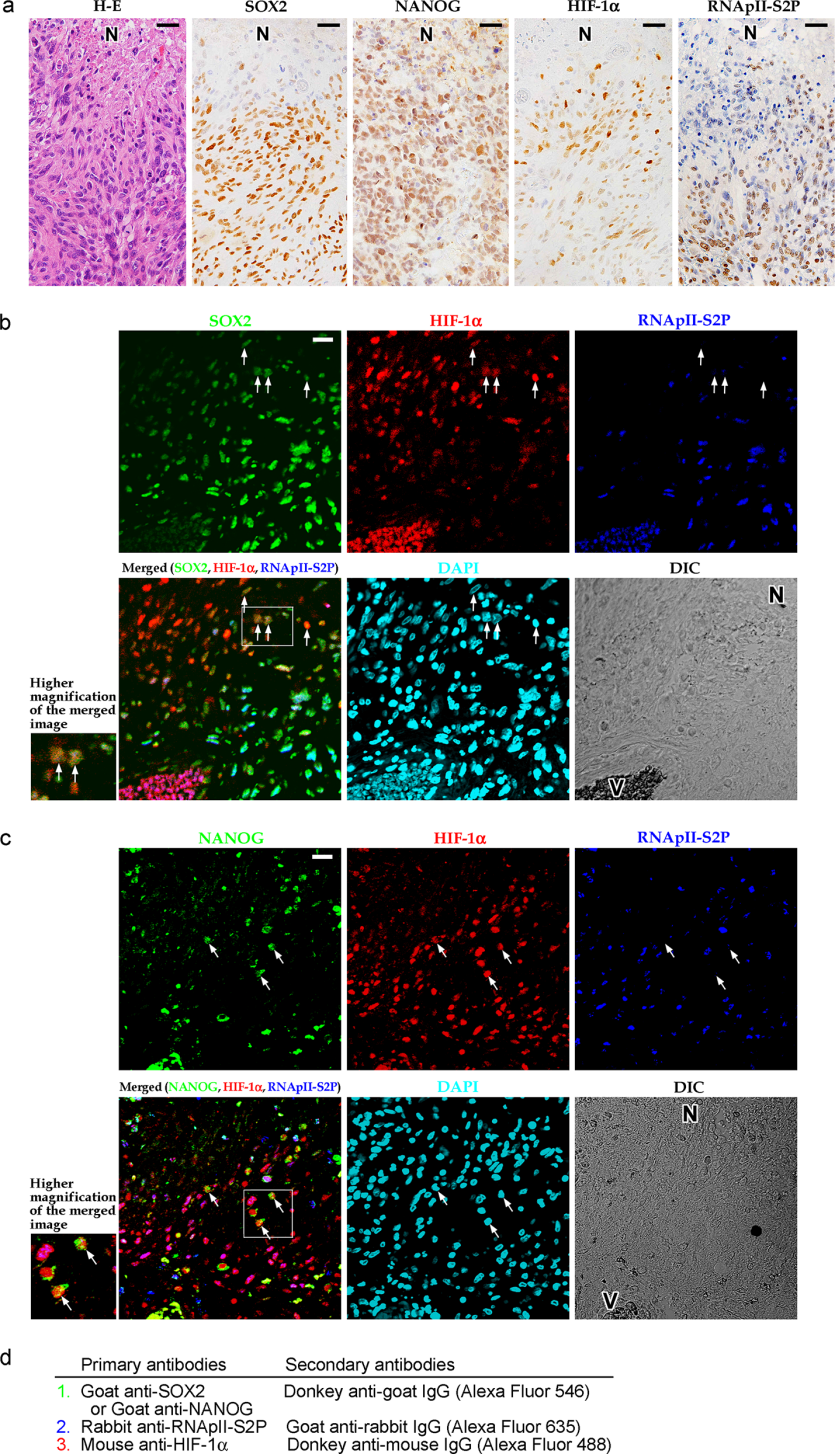
Tumor cells with a SOX2^+^ (or NANOG^+^) HIF-1α^+^ RNApII-S2P^-/low^ phenotype are found in human glioblastoma tissues. **a:** Hematoxylin-eosin (H-E) staining and single-color immunostaining for SOX2, NANOG, HIF-1α, and RNApII-S2P (serine 2 phosphorylation of the C-terminal domain of RNA polymerase II) in tumor tissue from a representative case of glioblastoma. Suppression of RNApII-S2P immunoreactivity was noted around the necrotic area (N). **b:** Triple immunofluorescent staining for SOX2, HIF-1α, and RNApII-S2P. SOX2^+^ HIF-1α^+^ RNApII-S2P^-/low^ cells (arrows) were found around the necrotic area (N). **c:** Triple immunofluorescent staining for NANOG, HIF-1α, and RNApII-S2P. NANOG^+^ HIF-1α^+^ RNApII-S2P^-/low^ cells (arrows) were found around the necrotic area (N). Insets at lower left of the panels b and c show higher magnification of the boxed areas in the merged images. N, necrotic area; V, blood vessels; DAPI, 4′,6-diamidino-2-phenylindole (nuclear stain); DIC, differential interference contrast image. Scale bars, 25 μm. **d:** Summary of the staining methods used in b and c. Sections were incubated with goat anti-SOX2 (b) or anti-NANOG (c) antibody and then with Alexa Fluor 546-conjugated donkey anti-goat IgG secondary antibody. Next, rabbit anti-RNApII-S2P antibody and then Alexa Fluor 635-conjugated goat anti-rabbit IgG secondary antibody were applied. The sections were reacted with mouse anti-HIF-1α antibody and then with biotinylated donkey anti-mouse IgG secondary antibody and Alexa Fluor 488-conjugated streptavidin.

We attempted to identify HIF-1α-regulated quiescent stem-like tumor cells in astrocytoma tissues using the following immunophenotypes as indices: SOX2^+^ HIF-1α^+^ RNApII-S2P^-/low^ or NANOG^+^ HIF-1α^+^ RNApII-S2P^-/low^. First, the expression of SOX2, NANOG, HIF-1α, and RNApII-S2P in glioblastoma tissue was investigated by single-color immunohistochemistry (Fig 1A). SOX2 and NANOG were immunolocalized to the nuclei of tumor cells with high frequency. Nuclear immunoreactivity for HIF-1α was also detected in a significant number of tumor cells, and these cells were predominantly located adjacent to necrotic areas. The phosphorylation-specific antibody for RNApII-S2P also labeled most of the tumor cell nuclei, but the immunoreactivity was suppressed in tumor cells around necrotic areas. We also examined the expression of CA IX, another marker of cellular hypoxia. CA IX is known as a hypoxia-induced transmembrane enzyme that regulates microenvironmental pH [35, 36]. As shown in S1 Fig, HIF-1α^+^ cells and CA IX^+^ cells were similarly distributed in the zones around necroses.

As a preliminary experiment, to verify the appropriate detection of quiescence with the phosphorylation-specific antibody for RNApII-S2P, the distribution of RNApII-S2P^-/low^ cells was compared with that of Ki-67-negative (Ki-67^-^) cells. *In vitro*, both RNApII-S2P^-/low^ cells and Ki-67^-^ cells appeared under the culture condition inducing quiescence (serum starvation), but did not appear under the condition accelerating proliferation (S2A Fig). Under the *in vitro* quiescent condition, the frequency of RNApII-S2P^-/low^ cells was lower than that of Ki-67^-^ cells. A lower frequency of RNApII-S2P^-/low^ cells compared with that of Ki-67^-^ cells was also noted in glioblastoma tissues (S2B Fig), suggesting the more strict specificity of the RNApII-S2P^-/low^ phenotype to quiescent cells with suppressed transcription similar to that of tissue stem cells.

Subsequently, we performed triple immunofluorescent staining for SOX2, HIF-1α, and RNApII-S2P, and found a small number of tumor cells showing a SOX2^+^ HIF-1α^+^ RNApII-S2P^-/low^ phenotype in the vicinity of necrotic areas (Fig 1B). The viability of these SOX2^+^ HIF-1α^+^ RNApII-S2P^-/low^ cells was confirmed by quadruple immunofluorescent staining, demonstrating their RNApII-S5P^+^ phenotype (S3 Fig). By triple immunofluorescent staining in which NANOG was substituted for SOX2, a similar small subpopulation of tumor cells with a NANOG^+^ HIF-1α^+^ RNApII-S2P^-/low^ phenotype was detected in the vicinity of necrotic areas (Fig 1C). When we counted the cells in the immunofluorescent images of the tumor tissues around necrotic areas, SOX2^+^ HIF-1α^+^ RNApII-S2P^-/low^ cells and NANOG^+^ HIF-1α^+^ RNApII-S2P^-/low^ cells accounted for 0–6.3% and 0–4.7% of the cells in the microscopic field, respectively.

### SOX2^+^ HIF-1α^+^ RNApII-S2P^-/low^ and NANOG^+^ HIF-1α^+^ RNApII-S2P^-/low^ tumor cells are associated with large ischemic necroses and found exclusively in glioblastoma tissues

**Fig 2:**
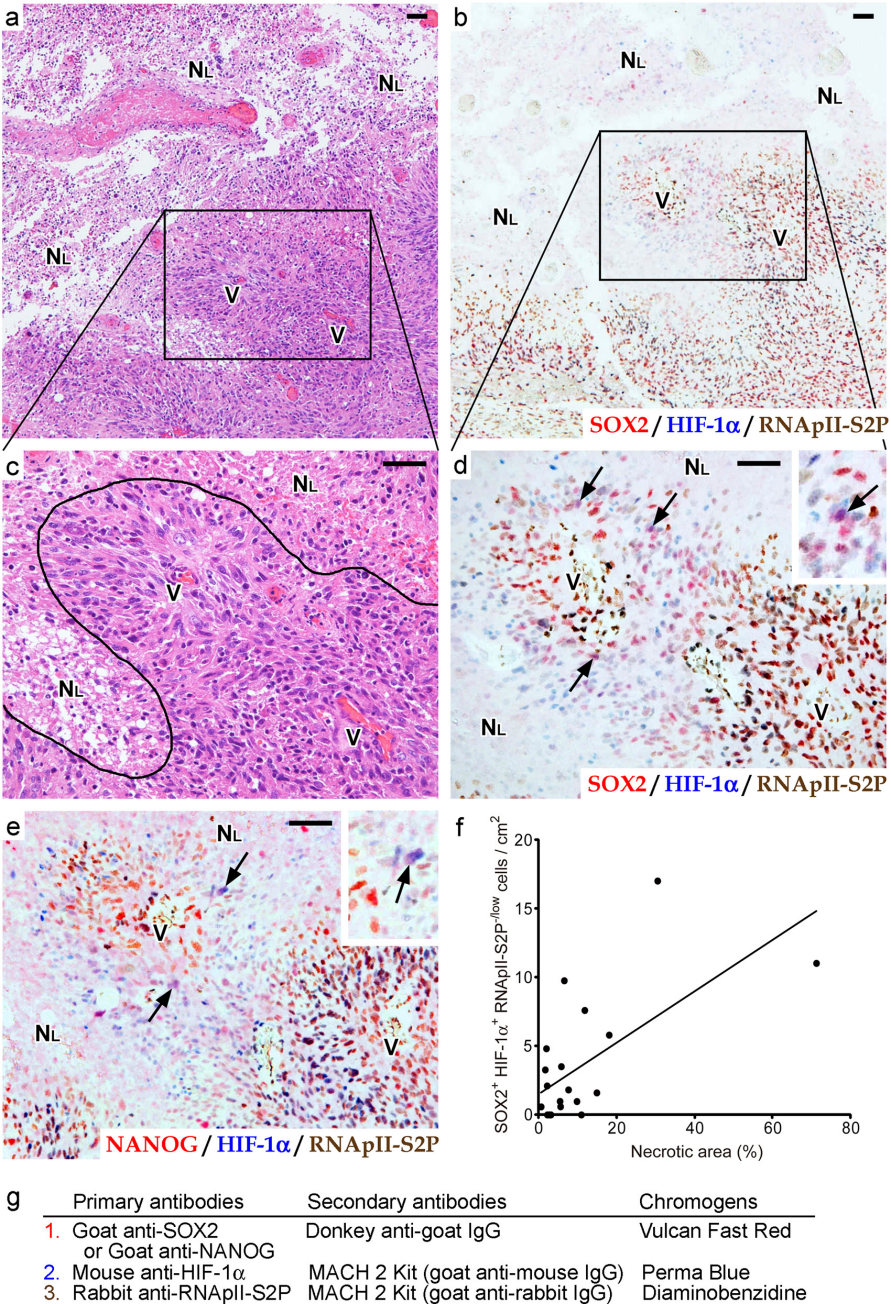
Detection of SOX2^+^ (or NANOG^+^) HIF-1α^+^ RNApII-S2P^-/low^ tumor cells by multi-color chromogenic immunostaining in glioblastoma tissues reveals that these cells are associated with large ischemic necroses. **a-e:** Serial sections of glioblastoma tissue around a large ischemic necrosis. H-E staining (a, c), triple staining for SOX2/HIF-1α/RNApII-S2P (b, d) or NANOG/HIF-1α/RNApII-S2P (e) are shown (SOX2 or NANOG, red; HIF-1α, blue; RNApII-S2P, brown). Boxes in a and b indicate the magnified areas in c and d, respectively. A curved line in c represents the border between a large ischemic necrosis and viable tumor tissue. Arrows in d and e mark SOX2^+^ HIF-1α^+^ RNApII-S2P^-/low^ cells and NANOG^+^ HIF-1α^+^ RNApII-S2P^-/low^ cells, respectively. Insets in d and e show higher magnifications of these cells (arrows). N_L_, large ischemic necrosis; V, blood vessels. Scale bars, 50 μm. **f:** Correlation between the relative area of necrosis and the frequency of SOX2^+^ HIF-1α^+^ RNApII-S2P^-/low^ cells in 21 glioblastoma cases. Line indicates the regression line (*y* = 0.188*x* + 1.46, Pearson’s correlation coefficient *r* = 0.64). **g:** Summary of the staining methods used in b and d–f. Sections were incubated with goat anti-SOX2 or anti-NANOG antibody and then with alkaline phosphatase (AP)-conjugated donkey anti-goat IgG secondary antibody, and color was developed with Vulcan Fast Red. After denaturing, mouse anti-HIF-1α antibody and rabbit anti-RNApII-S2P antibody were applied, and then the sections were reacted with MACH 2 Double Stain 1 (a secondary antibody cocktail of AP-conjugated anti-mouse IgG and horseradish peroxidase-conjugated anti-rabbit IgG antibodies). Color was developed with Perma Blue/AP for HIF-1α and diaminobenzidine for RNApII-S2P.

Since dark-field immunofluorescent microscopy is not suitable for characterizing the tissue distribution of SOX2^+^ HIF-1α^+^ RNApII-S2P^-/low^ and NANOG^+^ HIF-1α^+^ RNApII-S2P^-/low^ cells in various clinical samples, we switched our method from fluorescent to light-field chromogenic triple immunostaining (Fig 2). SOX2, HIF-1α, and RNApII-S2P were visualized with Vulcan Fast Red (bright red), Perma Blue (bright blue), and DAB (brown), respectively, and therefore nuclei of SOX2^+^ HIF-1α^+^ RNApII-S2P^-/low^ cells stained purple. To confirm that the combinations of overlaid chromogens could be distinguished from one another, a preliminary experiment was performed: identical triple immunostaining was applied to identify ER, PgR, and Ki-67 in a breast cancer tissue sample (S4 Fig). ER, PgR, and Ki-67 were visualized with Vulcan Fast Red, Perma Blue, and DAB, respectively. ER^+^ PgR^+^ Ki-67^-^ breast cancer cells were successfully recognized as nuclei with a purple color (S4E Fig), indicating that light-field chromogenic triple immunostaining using these chromogens is suitable for use in searching for SOX2^+^ (or NANOG^+^) HIF-1α^+^ RNApII-S2P^-/low^ cells in astrocytoma tissues.

**Fig 3:**
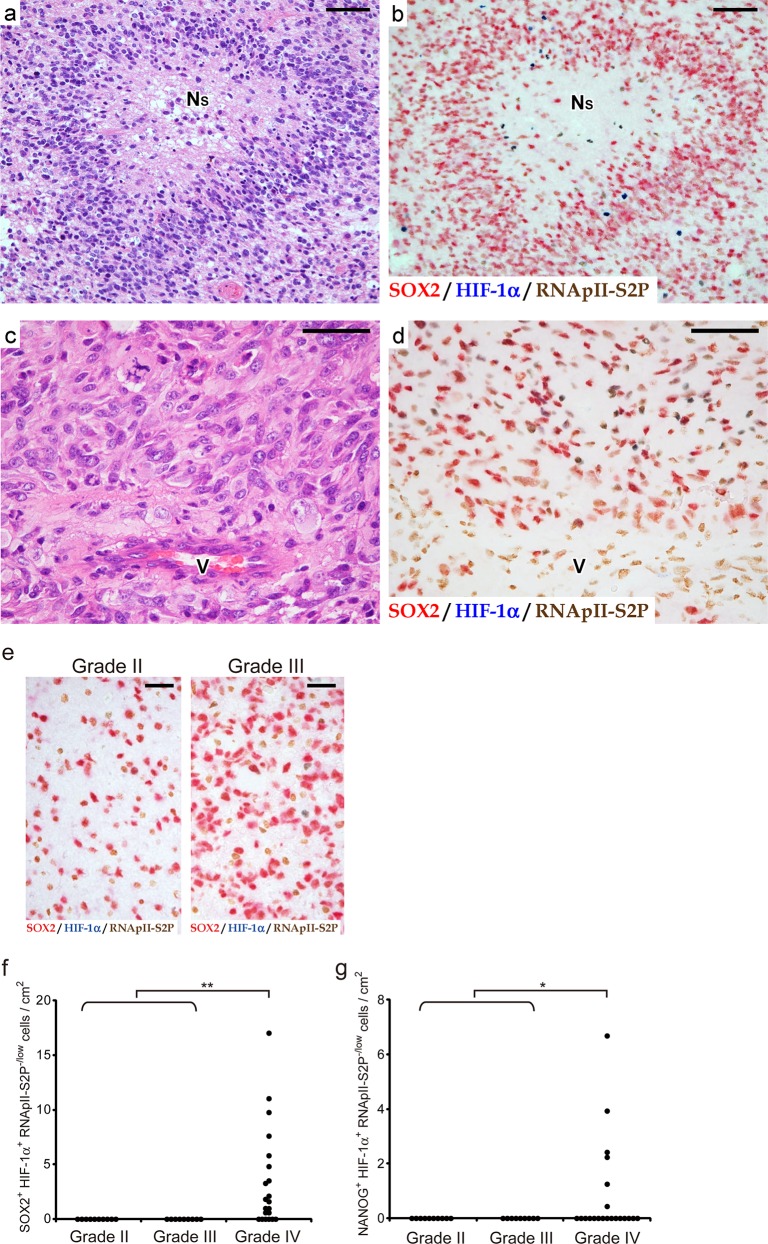
SOX2^+^ (or NANOG^+^) HIF-1α^+^ RNApII-S2P^-/low^ tumor cells are not found in zones around small pseudopalisading necroses or in areas showing no necrotic change. **These cells are found in glioblastomas (WHO grade IV), but not in diffuse astrocytomas (grade II) and anaplastic astrocytomas (grade III). a, b:** Serial sections of glioblastoma tissue containing small pseudopalisading necroses. **c, d:** Serial sections of glioblastoma tissue showing no necrotic changes. H-E staining (a, c) and triple immunostaining for SOX2/HIF-1α/RNApII-S2P (b, d) are shown. Although SOX2^+^ and/or HIF-1α^+^ tumor cells were found, they were RNApII-S2P^+^. Therefore, SOX2^+^ HIF-1α^+^ RNApII-S2P^-/low^ cells were not observed. N_S_, small pseudopalisading necrosis; V, blood vessel. Scale bars, 50 μm. **e:** Triple immunostaining for SOX2/HIF-1α/RNApII-S2P in astrocytomas of WHO grade II and III. No SOX2^+^ HIF-1α^+^ RNApII-S2P^-/low^ cells were observed. Scale bars, 25 μm. **f, g:** Frequency of SOX2^+^ HIF-1α^+^ RNApII-S2P^-/low^ cells (f) and NANOG^+^ HIF-1α^+^ RNApII-S2P^-/low^ cells (g) in cases of astrocytic tumors of WHO grade II–IV. *, *P* < 0.05; **, *P* < 0.01 (grade IV *vs*. combined group of grades II and III).

**Fig 4:**
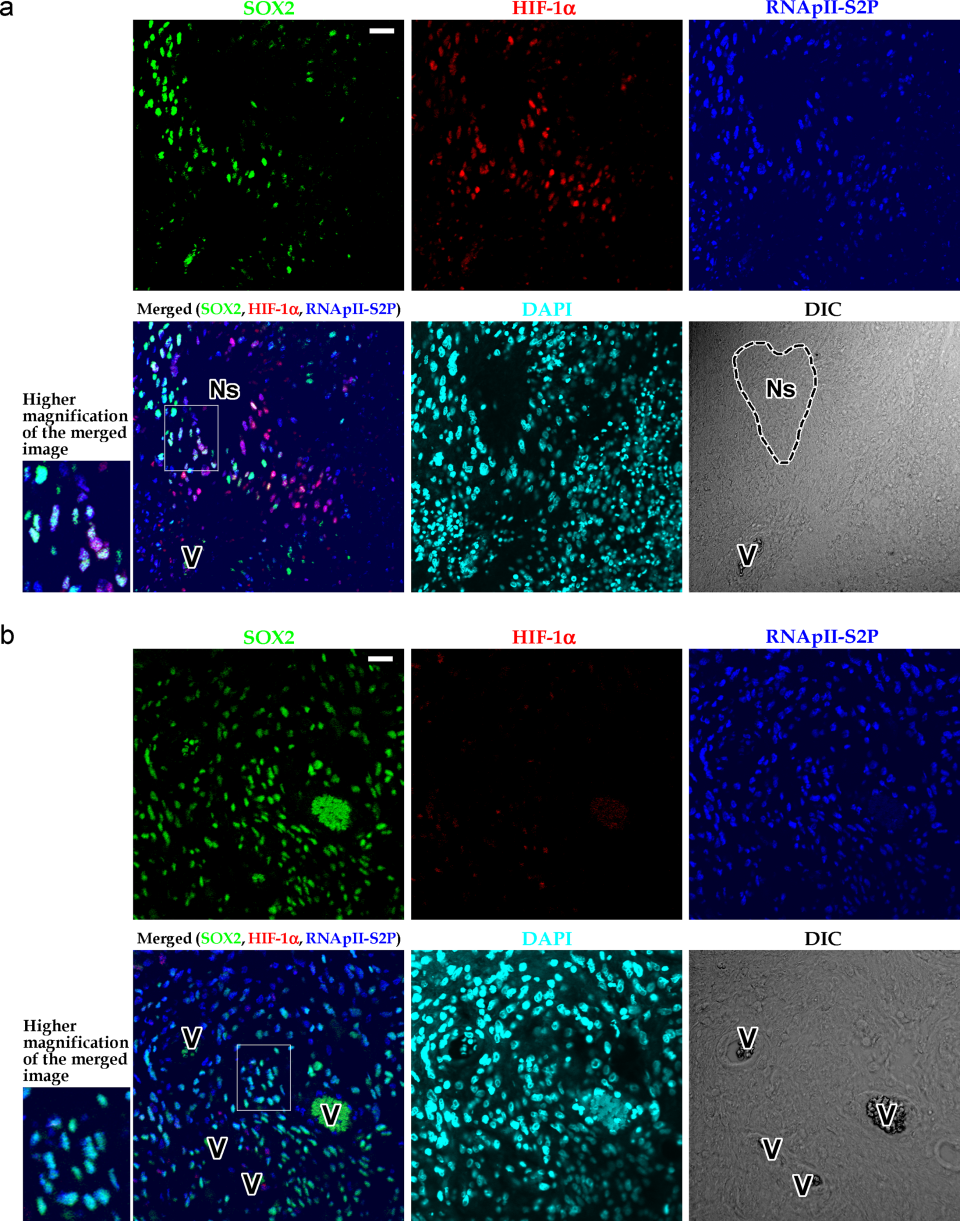
Triple immunofluorescent staining for SOX2, HIF-1α, and RNApII-S2P in zones around small pseudopalisading necroses or in areas showing no necrotic changes. **a:** Glioblastoma tissue containing small pseudopalisading necrosis. Although SOX2^+^ and/or HIF-1α^+^ tumor cells were found, they were RNApII-S2P^+^. Therefore, SOX2^+^ HIF-1α^+^ RNApII-S2P^-/low^ cells were not observed. **b:** Glioblastoma tissue showing no necrotic changes. SOX2^+^ HIF-1α^+^ RNApII-S2P^-/low^ cells were not found. Insets at lower left of the panels a and b show higher magnification of the boxed areas in the merged images. Ns, small pseudopalisading necrosis; V, blood vessels; DIC, differential interference contrast image. Scale bars, 25 μm.

As shown in Fig 2A–2D, SOX2^+^ HIF-1α^+^ RNApII-S2P^-/low^ cells were found, albeit at a low frequency, in glioblastoma tissues around large ischemic necroses. Similar findings were obtained when we substituted NANOG for SOX2 (Fig 2E). In our series of 21 glioblastoma cases, foci of large ischemic necrosis were observed in 20 cases, among which the existence of SOX2^+^ HIF-1α^+^ RNApII-S2P^-/low^ cells near large ischemic necroses was found in 16 cases. In the 4 cases without SOX2^+^ HIF-1α^+^ RNApII-S2P^-/low^ cells near a large ischemic necrosis, it is possible that these cells failed to be detected in the tissue sections used in this immunostaining because of the very low frequency of these cells. To further evaluate the association of these cells with large ischemic necroses in glioblastoma tissues, we measured the frequency of SOX2^+^ HIF-1α^+^ RNApII-S2P^-/low^ cells and compared it with the extent of necrosis in 21 cases of glioblastoma. The results demonstrated a significant correlation between the relative area of necrosis and the frequency of SOX2^+^ HIF-1α^+^ RNApII-S2P^-/low^ cells (Fig 2F; *y* = 0.188*x* + 1.46; Pearson’s correlation coefficient *r* = 0.64, *n* = 21, *P* < 0.01). In contrast, SOX2^+^ HIF-1α^+^ RNApII-S2P^-/low^ cells could neither be detected around small necroses with pseudopalisading of tumor cells, which is another type of necrosis in glioblastoma tissues [1, 37, 38] (Fig 3A and 3B), nor in the areas devoid of necrosis (Fig 3C and 3D). These findings were also confirmed by triple immunofluorescent staining (Fig 4). Using the immunofluorescent images, we counted 1200 cells in zones around small pseudopalisading necroses and 4600 cells in areas showing no necrotic change, but SOX2^+^ (or NANOG^+^) HIF-1α^+^ RNApII-S2P^-/low^ cells were not found. In addition, SOX2^+^ (or NANOG^+^) HIF-1α^+^ RNApII-S2P^-/low^ cells were not observed in grade II–III astrocytomas that lacked necrosis according to their diagnostic criteria, indicating that this subpopulation of tumor cells is specific to glioblastoma (Fig 3E–3G).

**Fig 5:**
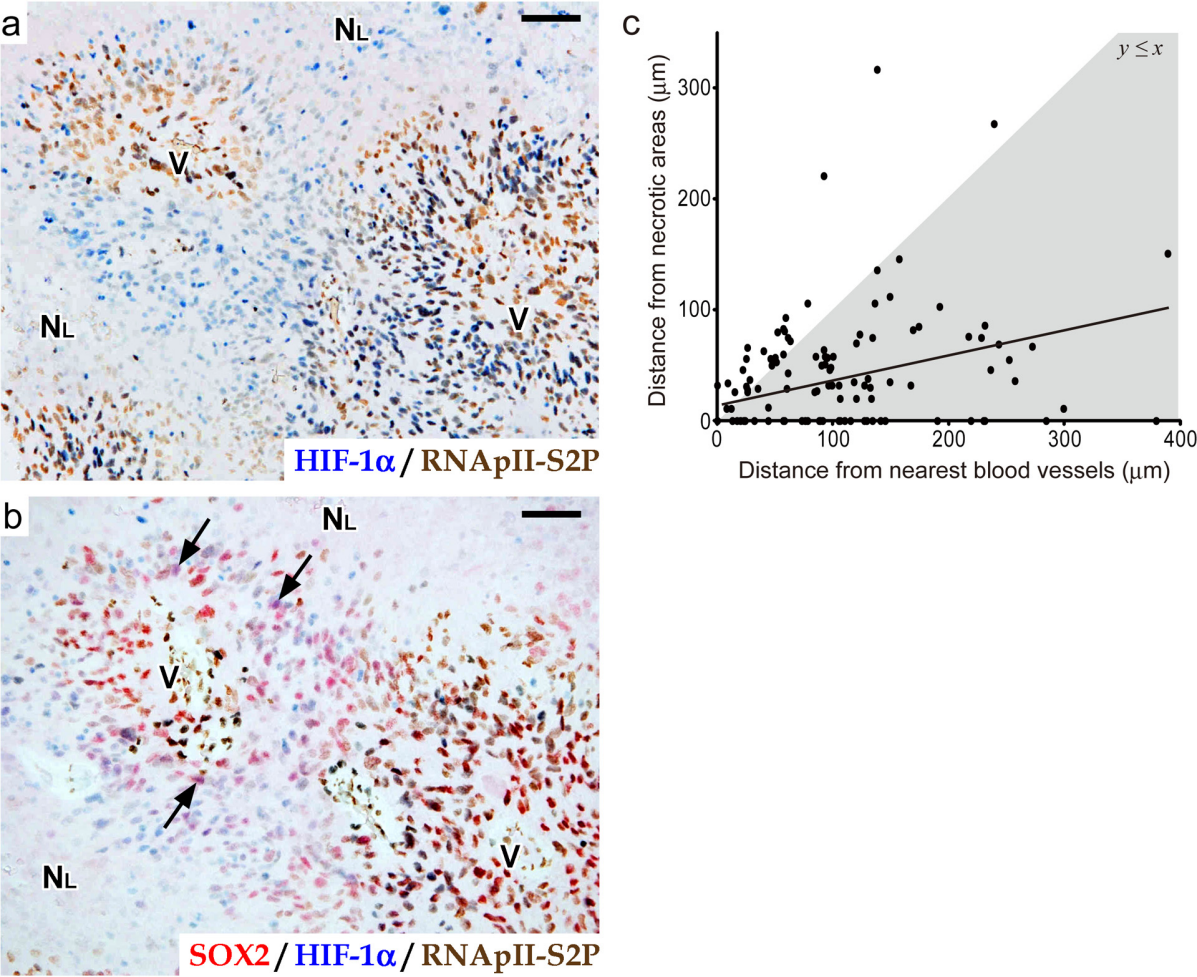
The localization of SOX2^+^ HIF-1α^+^ RNApII-S2P^-/low^ tumor cells is related to their distances from necrotic areas and blood vessels. **a**, **b:** Serial sections of glioblastoma tissue around a large ischemic necrosis. Double immunostaining for HIF-1α/RNApII-S2P (a) and triple immunostaining for SOX2/HIF-1α/RNApII-S2P (b) are shown (SOX2, red; HIF-1α, blue; RNApII-S2P, brown). Arrows in b indicate SOX2^+^ HIF-1α^+^ RNApII-S2P^-/low^ tumor cells. N_L_, large ischemic necrosis; V, blood vessels. Scale bars, 50 μm. **c:** Relationship between distances from nearest blood vessels and from necrotic areas in SOX2^+^ HIF-1α^+^ RNApII-S2P^-/low^ cells. The data from 135 SOX2^+^ HIF-1α^+^ RNApII-S2P^-/low^ cells found in 12 cases of glioblastoma are plotted. The line indicates the regression line (*y* = 0.205*x* + 17.4, *r* = 0.34). The gray region represents *y* ≤ *x*.

Double staining for HIF-1α and RNApII-S2P revealed that a significant number of HIF-1α^+^ RNApII-S2P^-/low^ tumor cells existed in the rather broad area around large ischemic necroses (blue nuclei in Fig 5A), in striking contrast to a small number of SOX2^+^ HIF-1α^+^ RNApII-S2P^-/low^ cells (purple nuclei in Fig 5B). This finding implied that only a small number of HIF-1α^+^ RNApII-S2P^-/low^ cells are selected to express stemness markers in response to a specific tissue microenvironment. In this context, it is noteworthy that SOX2^+^ (or NANOG^+^) HIF-1α^+^ RNApII-S2P^-/low^ cells were concentrated in the zones around necroses at a certain distance from the nearest blood vessels, where tumor cells are supposed to be exposed to moderate hypoxia and induced to express a moderate level of HIF-1α. To examine whether the emergence of SOX2^+^ HIF-1α^+^ RNApII-S2P^-/low^ cells was dependent on the distances from necrotic areas and blood vessels, we morphometrically measured the distances between SOX2^+^ HIF-1α^+^ RNApII-S2P^-/low^ cells and large ischemic necroses or the nearest blood vessels in the tissue sections. Fig 5C shows the data for 135 cells having the SOX2^+^ HIF-1α^+^ RNApII-S2P^-/low^ phenotype in glioblastomas, revealing that the localization of these cells tended to be determined by both of the parameters (*y* = 0.205*x* + 17.4; *r* = 0.34, *n* = 135, *P* < 0.01). The finding that most of the plots (80.7%) were distributed below the line *y = x* (gray region in Fig 5C) indicates that most SOX2^+^ HIF-1α^+^ RNApII-S2P^-/low^ cells were localized in the zone between large ischemic necroses and the nearest blood vessels, and were closer to the necrotic side than to the blood vessels. This suggests that the niche harboring HIF-1α-regulated quiescent stem-like tumor cells is present near large ischemic necroses (“peri-necrotic niche”), potentially under the influence of oxygen diffusion from blood vessels.

We also analyzed the frequency and localization of tumor cells showing immunophenotypes other than SOX2^+^ HIF-1α^+^ RNApII-S2P^-/low^ in the representative glioblastoma cases (*n* = 3). In contrast to the low frequency of SOX2^+^ HIF-1α^+^ RNApII-S2P^-/low^ cells (0–17 cells/cm^2^; Fig 3F), SOX2^+^ HIF-1α^-^ RNApII-S2P^+^ cells (1.9 × 10^5^–4.0 × 10^5^ cells/cm^2^) and SOX2^-^ HIF-1α^-^ RNApII-S2P^+^ cells (1.1 × 10^5^–2.7 × 10^5^ cells/cm^2^) were frequently found in perivascular areas and areas devoid of necrosis. The frequencies of SOX2^+^ HIF-1α^+^ RNApII-S2P^+^ cells (0–2.8 × 10^4^ cells/cm^2^) and SOX2^-^ HIF-1α^+^ RNApII-S2P^+^ cells (3.1 × 10^2^–2.1 × 10^4^ cells/cm^2^) were variable among cases, although these cells tended to be localized in the zones between necroses and nearest blood vessels. The frequencies of SOX2^-^ HIF-1α^+^ RNApII-S2P^-/low^ cells (1.4 × 10^1^–1.4 × 10^2^ cells/cm^2^) and SOX2^+^ HIF-1α^-^ RNApII-S2P^-/low^ cells (2.0 × 10^1^–1.1 × 10^2^ cells/cm^2^) were low, although they were higher than the frequency of SOX2^+^ HIF-1α^+^ RNApII-S2P^-/low^ cells. These RNApII-S2P^-/low^ cells in quiescent states showed the localization mainly in peri-necrotic areas, similarly to SOX2^+^ HIF-1α^+^ RNApII-S2P^-/low^ cells.

It has been reported that HIF-2α, another member of the HIF family, is implicated in the regulation of stem-like cells in glioma [23]. We compared the localization of SOX2^+^ HIF-1α^+^ RNApII-S2P^-/low^ cells with that of HIF-2α^+^ cells in serial sections of glioblastoma tissues (S5 Fig). HIF-2α^+^ cells were found both in areas around necroses and in perivascular areas. Only occasionally, HIF-2α^+^ cells were found in close spatial correlation with SOX2^+^ HIF-1α^+^ RNApII-S2P^-/low^ cells in the areas around large ischemic necroses (S5A and S5B Fig). However, the distributions of HIF-2α^+^ cells and SOX2^+^ HIF-1α^+^ RNApII-S2P^-/low^ cells were mostly different within peri-necrotic areas (S5C and S5D Fig) as well as within non-necrotic areas (S5E and S5F Fig), suggesting that HIF-2α is expressed in a subpopulation of tumor cells that are mostly different from SOX2^+^ HIF-1α^+^ RNApII-S2P^-/low^ cells.

### NANOG^+^ HIF-1α^+^ RNApII-S2P^-/low^ cells emerge in multicellular spheroids of glioblastoma cells in a hypoxia-dependent manner, and their emergence is associated with an enhanced sphere-forming activity

**Fig 6:**
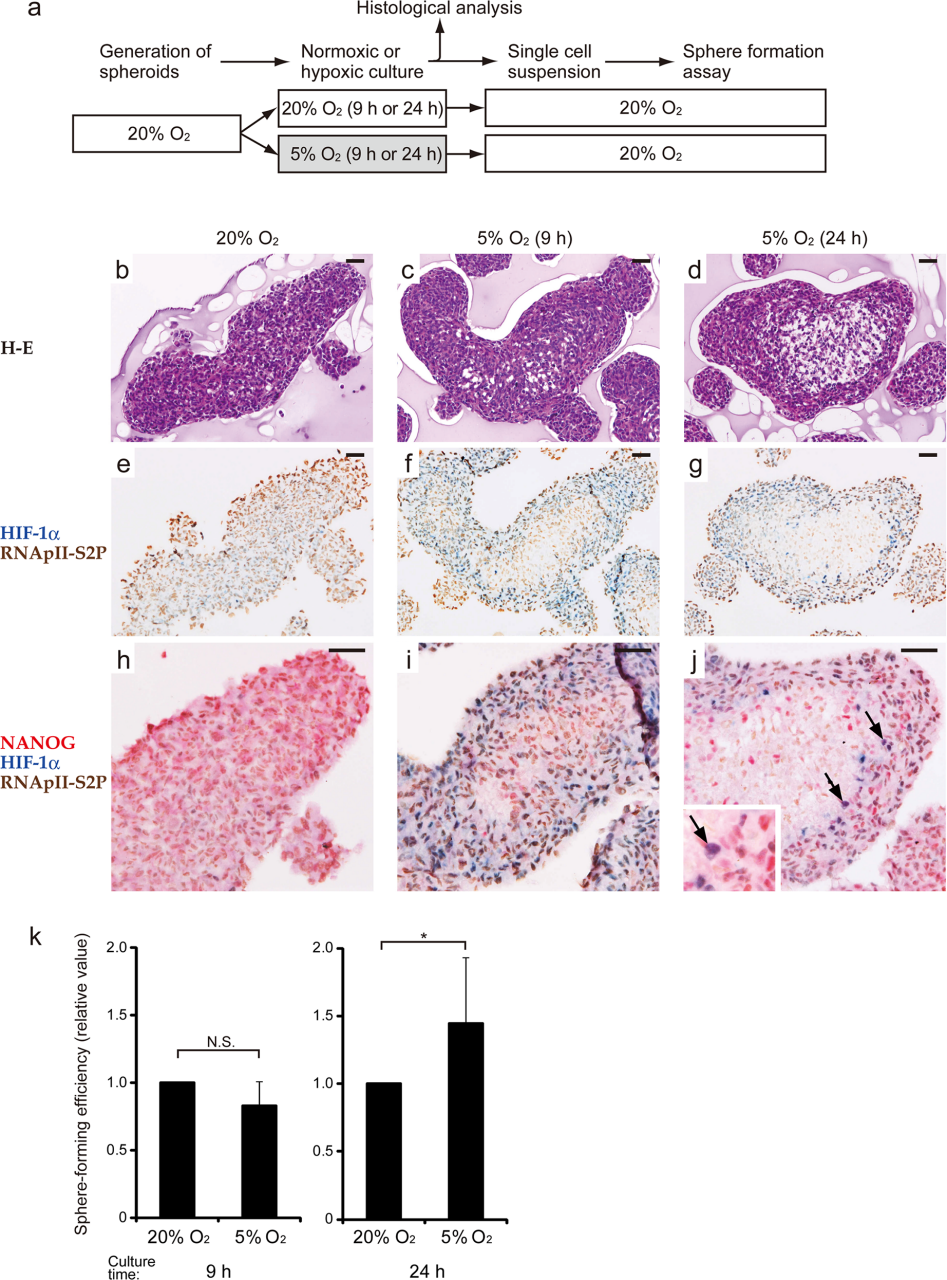
Significance of emergence of NANOG^+^ HIF-1α^+^ RNApII-S2P^-/low^ cells in spheroid cultures of T98G glioblastoma cells. **a:** Scheme of the experiments. Spheroids were generated under normoxic conditions (20% O_2_), and then divided into 2 groups (normoxic and hypoxic). After normoxic or hypoxic (5% O_2_) culture for 9 or 24 h, histological analysis and sphere formation assay under normoxic conditions were performed. Data for the normoxic and hypoxic groups sampled after the same culture time were compared. **b–j:** Histological analysis of the spheroids cultured under normoxic or hypoxic conditions. Since the spheroids of normoxic group cultured for 9 and 24 h showed similar results, only the spheroids cultured for 24 h are shown. H-E staining (b–d), chromogenic double immunostaining for HIF-1α/RNApII-S2P (e–g), and triple immunostaining for NANOG/HIF-1α/RNApII-S2P (h–j) are presented (NANOG, red; HIF-1α, blue; RNApII-S2P, brown). NANOG^+^ HIF-1α^+^ RNApII-S2P^-/low^ cells (purple cells) were detected in spheroids cultured in hypoxia for 24 h (j, arrows). The inset in j shows a higher magnification of these cells. These data are representative of at least 3 independent experiments. Scale bars, 50 μm. **k:** Sphere-forming efficiency of the cells derived from the spheroids of normoxic or hypoxic groups. Values are relative ones in which the results for the normoxic spheroids sampled after the same culture time were set to 1. The results are expressed as the mean ± SD of at least 7 independent experiments. N.S., not significant; *, *P* < 0.05.

Next, we established an *in vitro* model of the above-mentioned niche by culturing multicellular spheroids. The human glioblastoma cell line T98G was cultured under normoxia (20% O_2_) to generate spheroids, and then the spheroids were further cultured for 9 or 24 h under normoxic or hypoxic (5% O_2_) conditions (Fig 6A). Under normoxia, cells composing the spheroids were diffusely positive for RNApII-S2P, and negative or weakly positive for HIF-1α (Fig 6E). Up-regulation of HIF-1α was observed in the spheroids maintained under hypoxia for 9 as well as 24 h (Fig 6F and 6G). As for RNApII-S2P, almost all the constituent cells were RNApII-S2P^+^ following 9 h under hypoxia (Fig 6F), while RNApII-S2P^-/low^ cells appeared only in the central areas of spheroids after 24 h under hypoxia (Fig 6G). Triple immunostaining for NANOG, HIF-1α, and RNApII-S2P showed clearly that NANOG^+^ HIF-1α^+^ RNApII-S2P^-/low^ cells appeared exclusively in the spheroids after 24 h under hypoxia (Fig 6J). Interestingly, NANOG^+^ HIF-1α^+^ RNApII-S2P^-/low^ cells tended to be localized in the zone between the central core, which must be severely hypoxic, and the superficial zone, which is exposed to 5% O_2_, of the spheroids. This zone containing NANOG^+^ HIF-1α^+^ RNApII-S2P^-/low^ cells is considered a model corresponding to the “peri-necrotic niche” in glioblastoma tissues. We next triturated the spheroids under these 3 conditions to perform a sphere formation assay under normoxic conditions. The sphere-forming efficiency (SFE) of the spheroids after 9 h under hypoxia was comparable to that of normoxic controls, while cells obtained from the spheroids following 24 h under hypoxia showed a significantly (*P* < 0.05) higher SFE than that of normoxic controls (Fig 6K). These results demonstrate that the spheroids cultured under 5% O_2_ for 24 h gained an enhanced tumorigenic capacity, presumably by generating a moderately hypoxic niche harboring tumor cells of the NANOG^+^ HIF-1α^+^ RNApII-S2P^-/low^ phenotype.

**Fig 7:**
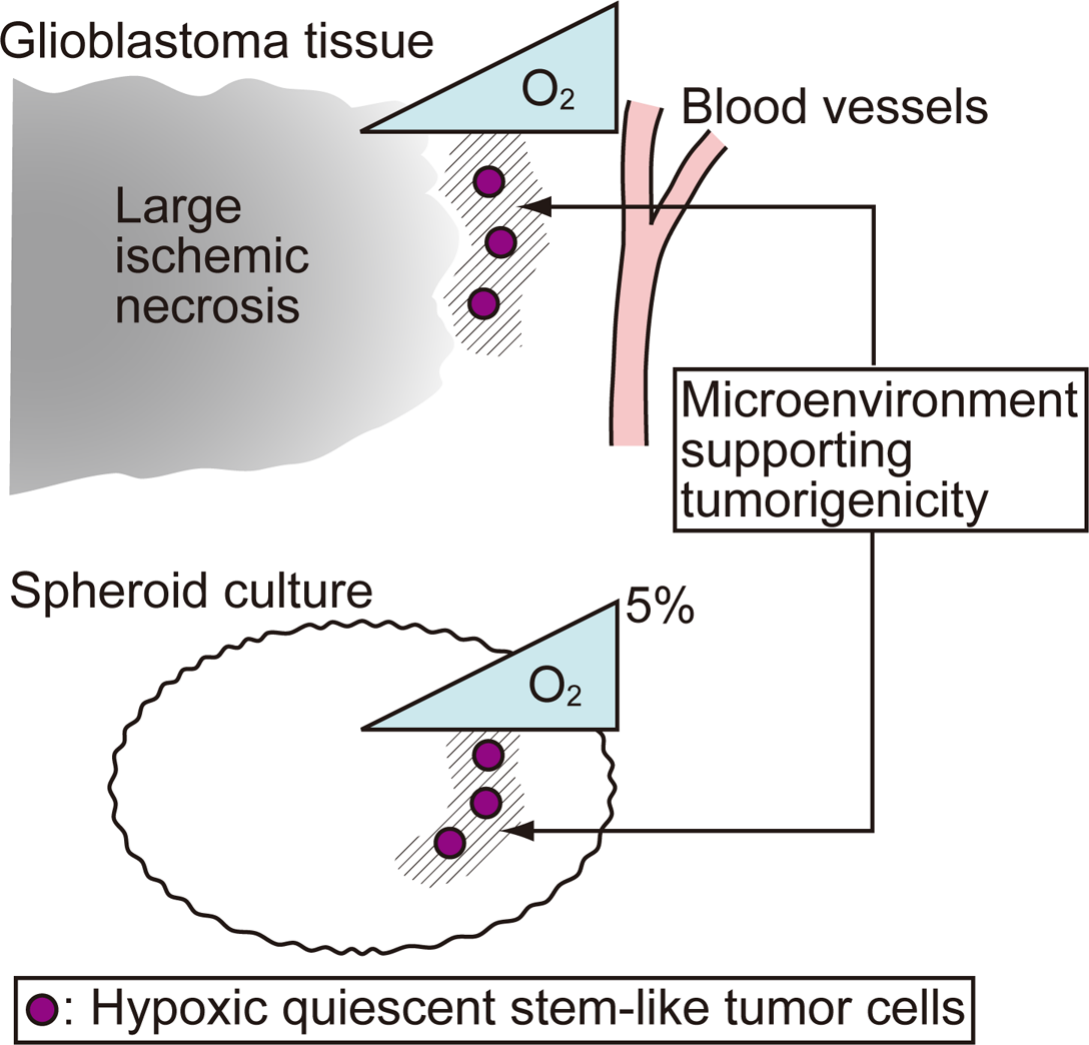
A schematic model illustrating the main findings of this study. The SOX2^+^ (or NANOG^+^) HIF-1α^+^ RNApII-S2P^-/low^ cells identified in this study represent hypoxic quiescent stem-like tumor cells, and the emergence of these cells is associated with an increased sphere-forming activity. Thus, the most common locations of these cells, namely, the neighborhood of large ischemic necroses in glioblastoma tissues (“peri-necrotic niche”) and the zone of intermediate depth from the surface of the spheroids cultured under 5% O_2_, indicate a microenvironment supporting tumorigenicity. Since a gradient of O_2_ concentration is present within the glioblastoma tissues and spheroids, this microenvironment is formed under an appropriate level of hypoxia.

Taken together, these data imply that the “peri-necrotic niche” harboring HIF-1α^+^ quiescent stem-like tumor cells in glioblastoma tissues can be generated in an intratumoral gradient of hypoxia and is associated with an enhanced tumorigenic capacity (Fig 7).

## Discussion

A niche harboring GSCs with a high long-term tumorigenic capacity would represent a target for new therapies to eradicate glioblastoma cells. In the context of the repopulation capacity of normal HSCs, we previously showed that the niche that maintains a moderate level of HIF-1α protein expression is essential for keeping HSCs in a quiescent state, and that these HIF-1α-regulated quiescent HSCs exhibit a high capacity for long-term repopulation after bone marrow transplantation [26]. Since normal tissue stem cells and CSCs tend to share common underlying mechanisms to control their stemness [39], we applied the concept of HIF-1α-regulated quiescence to GSCs in order to clarify whether a similar niche may play important roles in the pathophysiology of glioblastoma. In the present study, by combining clinical sample analyses with *in vitro* experiments, we characterized a niche harboring quiescent stem-like tumor cells of the SOX2^+^ HIF-1α^+^ RNApII-S2P^-/low^ or NANOG^+^ HIF-1α^+^ RNApII-S2P^-/low^ phenotypes, which are associated with an enhanced tumorigenic capacity. Within glioblastoma tissues, this niche was mapped to a location between large ischemic necroses and the nearest blood vessels, and was closer to the necrotic side than to the blood vessels. Since a gradient of oxygen concentration is present between the blood vessels, which have a relatively high oxygen concentration, and the foci of ischemic necrosis, which are under intense hypoxia (Fig 7), the zone containing SOX2^+^ (or NANOG^+^) HIF-1α^+^ RNApII-S2P^-/low^ cells may be under an appropriate degree of hypoxia for maintaining the expression of HIF-1α protein at a moderate level, which would maintain the tumor cells under a quiescent state and support a higher tumorigenic potential. The hypoxic activation of the HIF-1 pathway in this “peri-necrotic” zone was implied by our finding that HIF-1α, as well as another hypoxia-induced protein, CA IX, were expressed by tumor cells in this zone. This interpretation of the histological data was supported by our *in vitro* experiments with spheroids, which also have a gradient of oxygen concentration between the surface area (which has a relatively high oxygen concentration) and the central core (which is under severe hypoxia) (Fig 7). In spheroids cultured under normoxia (20% O_2_), NANOG^+^ HIF-1α^+^ RNApII-S2P^-/low^ cells could not be detected. When we decreased the oxygen concentration in the cell culture incubator from 20% to 5% without changing other culture conditions, NANOG^+^ HIF-1α^+^ RNApII-S2P^-/low^ cells appeared in spheroids after 24 h. Spheroids subjected to hypoxia for 24 h had areas containing quiescent (RNApII-S2P^-/low^) cells in their centers, and NANOG^+^ HIF-1α^+^ RNApII-S2P^-/low^ cells emerged in the zone between the surface and the central core of spheroids, and were closer to the central core than to the surface. It is noteworthy that NANOG^+^ HIF-1α^+^ RNApII-S2P^-/low^ cells could not be detected in spheroids under 9 h hypoxia, in which a clear-cut area of RNApII-S2P^-/low^ cells did not develop. The SFE of spheroids under 24 h hypoxia, which contained NANOG^+^ HIF-1α^+^ RNApII-S2P^-/low^ cells, was enhanced, while spheroids under 9 h hypoxia, which lacked these cells, showed no significant increase in SFE. Thus, the up-regulation of HIF-1α itself is not sufficient for glioblastoma cells to obtain a higher tumorigenic capacity, and the additional induction of quiescence appears to be crucial for this raised tumorigenic capacity to occur. The biological importance of SOX2^+^ (or NANOG^+^) HIF-1α^+^ RNApII-S2P^-/low^ tumor cells was also confirmed by our finding that these cells were found exclusively in glioblastoma tissues but not in grade II–III astrocytoma tissues. Therefore, the presence of the niche harboring these cells might be one aspect of the microenvironmental heterogeneity that is a distinctive feature of glioblastoma. Taken together, it can be concluded that an appropriately hypoxic microenvironment around large ischemic necroses (the “peri-necrotic niche”) in glioblastoma provides a niche for stem-like tumor cells associated with high tumorigenic capacity (Fig 7).

In glioblastoma tissues, there are 2 types of necroses, large ischemic necrosis and small pseudopalisading necrosis [1, 37, 38]. The extent of large ischemic necroses has been described as an adverse prognostic factor [40, 41]. This clinicopathological relevance supports our immunohistochemical data, which highlight the importance of large ischemic necroses for providing the “peri-necrotic niche”. SOX2^+^ (or NANOG^+^) HIF-1α^+^ RNApII-S2P^-/low^ cells were found in the zones around large ischemic necroses, but not in those around small pseudopalisading necroses. This difference between the 2 types of necroses might reflect the distinct mechanisms underlying their occurrence. Brat *et al*. proposed that cells around pseudopalisading necroses are composed of tumor cells actively migrating away from a hypoxic focus and showing a higher rate of apoptosis [42], which is in striking contrast to the cells around large ischemic necroses. In our present study, the cell-cycle status of peri-necrotic tumor cells appeared to be different between the 2 types of necroses. Tumor cells around large ischemic necroses were RNApII-S2P^-/low^, while cells around small pseudopalisading necroses were RNApII-S2P^+^. Thus, although there was a previous report showing that areas around pseudopalisading necroses contain cells positive for CD133, a putative marker for GSCs [43], it is reasonable to consider that the areas around small pseudopalisading necroses are distinct from the zones around large ischemic necroses that provide a niche for quiescent stem-like tumor cells.

Our *in vitro* data obtained from the analysis of spheroids revealed a close association between the microenvironment-dependent quiescence of glioblastoma cells and an enhanced tumorigenic potential. This implies that glioblastoma cells are flexible in that they become quiescent in hypoxic spheroids but begin to proliferate actively and form tumor spheres efficiently in response to microenvironmental changes. Quiescence is one of the states of the G_0_ phase of the cell cycle. Quiescent cells must be distinct from terminally differentiated cells and senescent cells, both of which irreversibly exited from the cell cycle. Quiescence is characterized by the ability of cells to re-enter the cell cycle in response to appropriate stimuli [14]. These quiescent cells can be distinguished from terminally differentiated or senescent cells by their characteristic phosphorylation pattern of RNA polymerase II (RNApII-S2P^-/low^ and RNApII-S5P^+^), indicating that transcription is initiated but transcriptional elongation is suppressed [14]. Our immunohistochemical finding that only a small subpopulation of Ki-67^-^ glioblastoma cells showed an RNApII-S2P^-/low^ phenotype reflects the difference in the type of G_0_ phase between the Ki-67^-^ cells and RNApII-S2P^-/low^ cells. The former includes not only quiescent cells but also terminally differentiated cells and senescent cells, whereas the latter is specific for quiescent cells. Hypoxia-induced cell-cycle arrest could reportedly be mediated by the action of HIF-1α through the disruption of c-Myc function, suppression of cyclin D expression, and induction of p21^Cip1^ [44, 45]. In contrast, little is known about the mechanisms by which HIF-1α concurrently confers stemness on cells.

Accumulating studies have highlighted the perivascular areas in tumors as a candidate for a GSC niche [12, 46–48]. The importance of the perivascular niche for GSCs is supported by the idea that GSCs must share the same mechanisms as normal neural stem cells, which reside around blood vessels, to promote and maintain their stemness under the influence of paracrine factors secreted by vascular endothelial cells and/or perivascular cells, for example, nitric oxide [46], sonic hedgehog [48], and osteopontin [47]. Since perivascular areas are relatively abundant in oxygen and nutrients, these areas are thought to be the microenvironment suitable for cell proliferation. On the other hand, the peri-necrotic niche specified in our study is characterized by a paucity of oxygen and nutrients, which could activate hypoxia-dependent pathways and consequently confer quiescence as well as stemness on tumor cells. Thus, it is probable that stem-like tumor cells in the perivascular niche are closely related to the phenotype of cells in the proliferative state, while those in the peri-necrotic niche show the phenotype of dormant long-term stem-like cells that can be activated to undergo proliferation upon microenvironmental changes.

While several lines of evidence have indicated that HIF-1α mediates the hypoxia-induced promotion of stemness in glioma cells [24, 25], the implication of HIF-2α in the biology of GSCs has also been demonstrated. Li *et al*. [23] reported that HIF-2α is preferentially expressed by CD133^+^ putative GSCs under both hypoxic and normoxic conditions, whereas HIF-1α is induced under hypoxia not only in CD133^+^ but also in CD133^-^ glioma cells. Both HIF-1α and HIF-2α have been shown to be crucially involved in the promotion of *in vitro* sphere formation, cell growth, and the survival of CD133^+^ glioma cells [23]. To discuss the roles of HIF-2α in the stemness and quiescence of glioblastoma cells within the peri-necrotic niche, which is the focus of our study, we compared the localization of HIF-2α^+^ cells with that of SOX2^+^ HIF-1α^+^ RNApII-S2P^-/low^ cells. Consistent with the previous report [23], we observed fewer HIF-2α^+^ cells than HIF-1α^+^ cells, and these cells were observed both in areas around necroses and in perivascular areas. The distributions of HIF-2α^+^ cells and SOX2^+^ HIF-1α^+^ RNApII-S2P^-/low^ cells overlapped only occasionally in the areas around large ischemic necroses, and the overall distribution patterns of these 2 subpopulations of tumor cells were essentially different. The perivascular localization of HIF-2α^+^ cells appeared to be consistent with the previous finding that HIF-2α, in contrast to HIF-1α, was expressed by CD133^+^ glioma cells under normoxia or mild hypoxia [23]. In the context of hypoxia-induced quiescence, an opposing function of HIF-2α to that of HIF-1α is suggested by reports showing that HIF-2α promotes proliferation under hypoxia through c-Myc activation or other mechanisms [49, 50]. Thus, we speculate that SOX2^+^ HIF-1α^+^ RNApII-S2P^-/low^ cells and HIF-2α^+^ cells might play different roles in the biology of glioblastoma. Although further studies are needed to clarify precisely how HIF-1α and HIF-2α share their contributions to quiescence and tumorigenicity in glioblastomas, it might be true that they are differently used to regulate the behavior of stem-like tumor cells including quiescence, proliferation, and so forth.

In summary, the present study highlights the intratumoral areas around large ischemic necroses as a niche that provides an appropriate microenvironment for glioblastoma cells to acquire quiescence and an enhanced tumorigenic potential. This “peri-necrotic niche” would be a promising target for new therapies to eradicate glioblastoma cells and overcome the therapeutic resistance of glioblastomas.

## Supporting Information

S1 Fig Expression of HIF-1α and carbonic anhydrase IX (CA IX) in glioblastoma tissue. Single-color immunostaining for HIF-1α (a) and CA IX (b) in serial sections of a representative glioblastoma tissue. The inset in b shows a higher magnification of CA IX^+^ tumor cells displaying membrane staining. N, necrotic area; V, blood vessels. Scale bars, 50 μm.
(JPG)

S2 Fig Comparison between RNApII-S2P^-/low^ cells and Ki-67^-^ cells. **a:** Regulation of Ki-67 and RNApII-S2P during proliferation and quiescence in T98G glioblastoma cells. T98G cells were grown in culture medium containing 10% (v/v) fetal bovine serum (FBS), were induced to become quiescent by serum starvation in medium supplemented with 0.5% (v/v) FBS for 14 days, and then were re-stimulated by being split 1:5 into new medium containing 10% (v/v) FBS and cultured for 3 days. The cells were detached from dishes with trypsin-EDTA solution, fixed in 10% (v/v) neutral buffered formalin, and centrifuged. Paraffin sections of the pellet were cut, and expression of Ki-67 and RNApII-S2P was examined by single (brown; a_1_, a_2_, a_4_, a_5_, a_7_, a_8_) or double immunostaining (Ki-67, brown; RNApII-S2P, red; a_3_, a_6_, a_9_). Hematoxylin (blue) was used as a nuclear stain. Ki-67^-^ RNApII-S2P^-/low^ cells (blue cells in the double stained sections) emerged only in the quiescent condition (a_6_, arrows). Scale bar, 10 μm. **b:** Single-color immunostaining for Ki-67 (b_1_) and RNApII-S2P (b_2_) in serial sections of glioblastoma tissue. Ki-67^-^ tumor cells were frequently found, whereas only a few RNApII-S2P^-/low^ cells (arrows) were observed around necrotic area. N, necrotic area; V, blood vessels. Scale bars, 50 μm.
(JPG)

S3 Fig Quadruple immunofluorescent staining for SOX2, HIF-1α, RNApII-S2P, and RNApII-S5P. SOX2^+^ HIF-1α^+^ RNApII-S2P^-/low^ cells (arrows) are positive for RNApII-S5P. As shown in the table (bottom), sections were incubated with goat anti-SOX2 antibody and then with Alexa Fluor 633-conjugated donkey anti-goat IgG secondary antibody. Next, rabbit anti-RNApII-S2P antibody and then Alexa Fluor 405-conjugated goat anti-rabbit IgG secondary antibody were applied. The sections were reacted with mouse anti-HIF-1α antibody and then with biotinylated donkey anti-mouse IgG secondary antibody and Alexa Fluor 488-conjugated streptavidin. Finally, rabbit anti-RNApII-S5P antibody directly labeled with Zenon Alexa Fluor 546 was applied. N, necrotic area; V, blood vessels; DIC, differential interference contrast image. Scale bar, 25 μm.
(JPG)

S4 Fig Chromogenic triple immunostaining for estrogen receptor (ER), progesterone receptor (PgR), and Ki-67 in breast cancer tissue to verify the triple immunostaining detection method. **a:** Hematoxylin-eosin (H-E) staining. **b–e:** Immunostaining of serial sections. Single immunohistochemistry for ER (b), PgR (c), and Ki-67 (d), and triple immunostaining for ER/PgR/Ki-67 (e) are shown (ER, red; PgR, blue; Ki-67, brown). In triple immunostaining (e), ER^+^ PgR^-^ Ki-67^-^ cells were stained red (short arrow), ER^-^ PgR^+^ Ki-67^-^ cells were stained blue (black arrowhead), ER^+^ PgR^+^ Ki-67^-^ cells were stained purple (long arrow), and Ki-67^+^ cells were stained brown (white arrowhead). These colors are easily distinguishable. Scale bars, 25 μm. **f:** Summary of the staining methods used in e. Sections were incubated with mouse anti-ER antibody and then with alkaline phosphatase (AP)-conjugated donkey anti-mouse IgG secondary antibody, and color was developed with Vulcan Fast Red. After denaturing, mouse anti-PgR antibody and rabbit anti-Ki-67 antibody were applied, and then the sections were reacted with MACH 2 Double Stain 1 (a secondary antibody cocktail of AP-conjugated anti-mouse IgG and horseradish peroxidase-conjugated anti-rabbit IgG antibodies). Color was developed with Perma Blue/AP for PgR and diaminobenzidine for Ki-67.
(JPG)

S5 Fig Localization of SOX2^+^ HIF-1α^+^ RNApII-S2P^-/low^ cells and HIF-2α^+^ cells in glioblastoma tissue. Triple immunostaining for SOX2/HIF-1α/RNApII-S2P (a, c, and e) and single immunostaining for HIF-2α (b, d, and f) are shown. **a, b:** Serial sections of an area around a large ischemic necrosis (N_L_). HIF-2α^+^ cells (black arrowheads in b) were found near SOX2^+^ HIF-1α^+^ RNApII-S2P^-/low^ cells (arrows in a), but this finding was only occasionally observed. HIF-2α^+^ cells were also found in perivascular areas (white arrowhead in b), whereas SOX2^+^ HIF-1α^+^ RNApII-S2P^-/low^ cells were not detected in these areas. **c, d:** Serial sections of another area around a large ischemic necrosis. HIF-2α^+^ cells were observed (arrowheads in d) whereas no SOX2^+^ HIF-1α^+^ RNApII-S2P^-/low^ cells were found in this location (c). **e, f:** Serial sections of an area devoid of necrosis. HIF-2α^+^ cells were found near blood vessels (arrowheads in f), but no SOX2^+^ HIF-1α^+^ RNApII-S2P^-/low^ cells were observed in this area (e). N_L_, large ischemic necrosis; V, blood vessels. Scale bars, 50 μm.
(JPG)
