# Supplementary figures and images for: Histological Characterization of the Tumorigenic “Peri-Necrotic Niche” Harboring Quiescent Stem-Like Tumor Cells in Glioblastoma
Source: PLoS One. 2016 Jan 22;11(1):e0147366. doi: 10.1371/journal.pone.0147366 (PMC4723051; doi:10.1371/journal.pone.0147366)

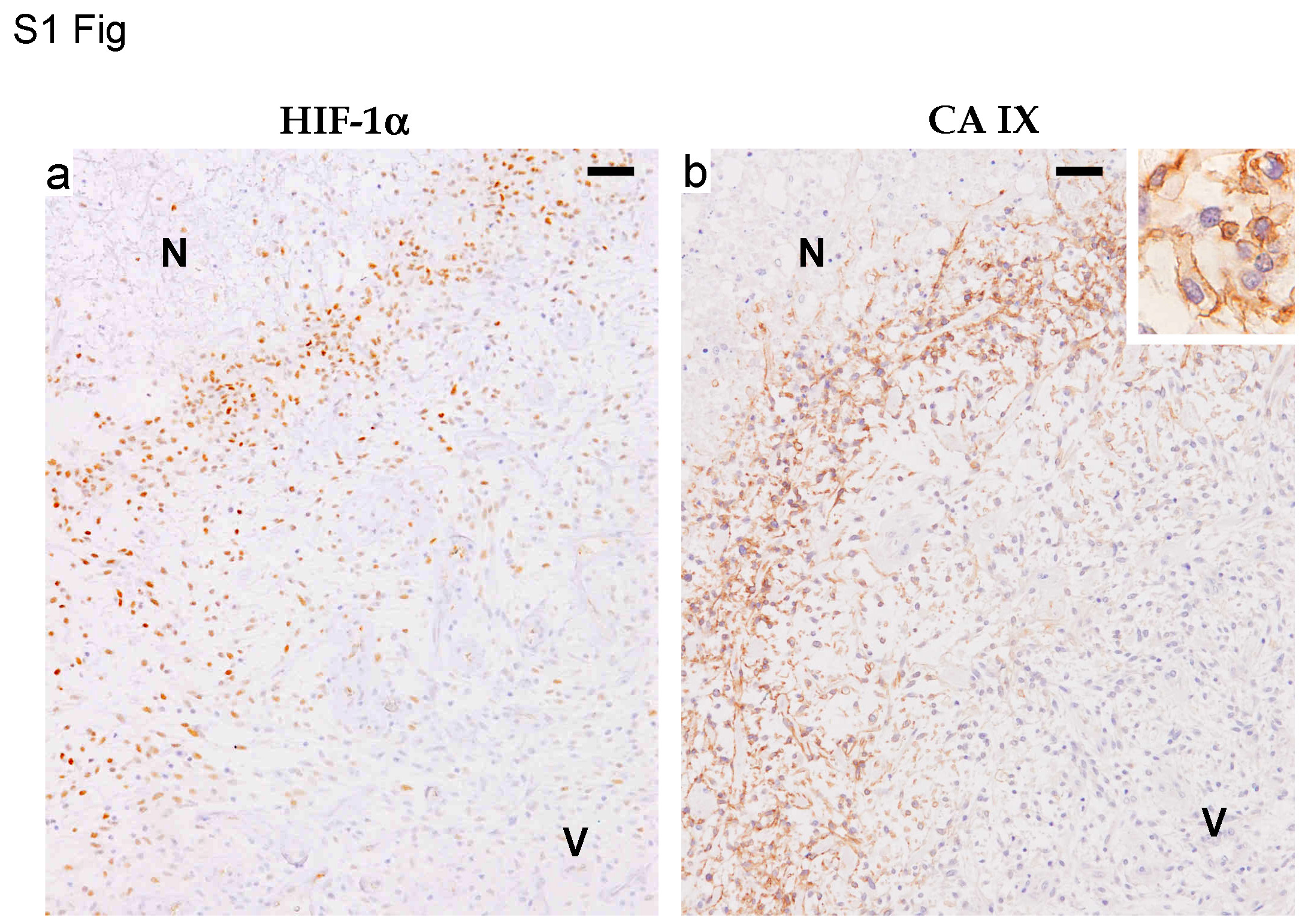

Supplement: S1 Fig — Single-color immunostaining for HIF-1α (a) and CA IX (b) in serial sections of a representative glioblastoma tissue. The inset in b shows a higher magnification of CA IX+ tumor cells displaying membrane staining. N, necrotic area; V, blood vessels. Scale bars, 50 μm. (JPG) [file pone.0147366.s001.jpg]

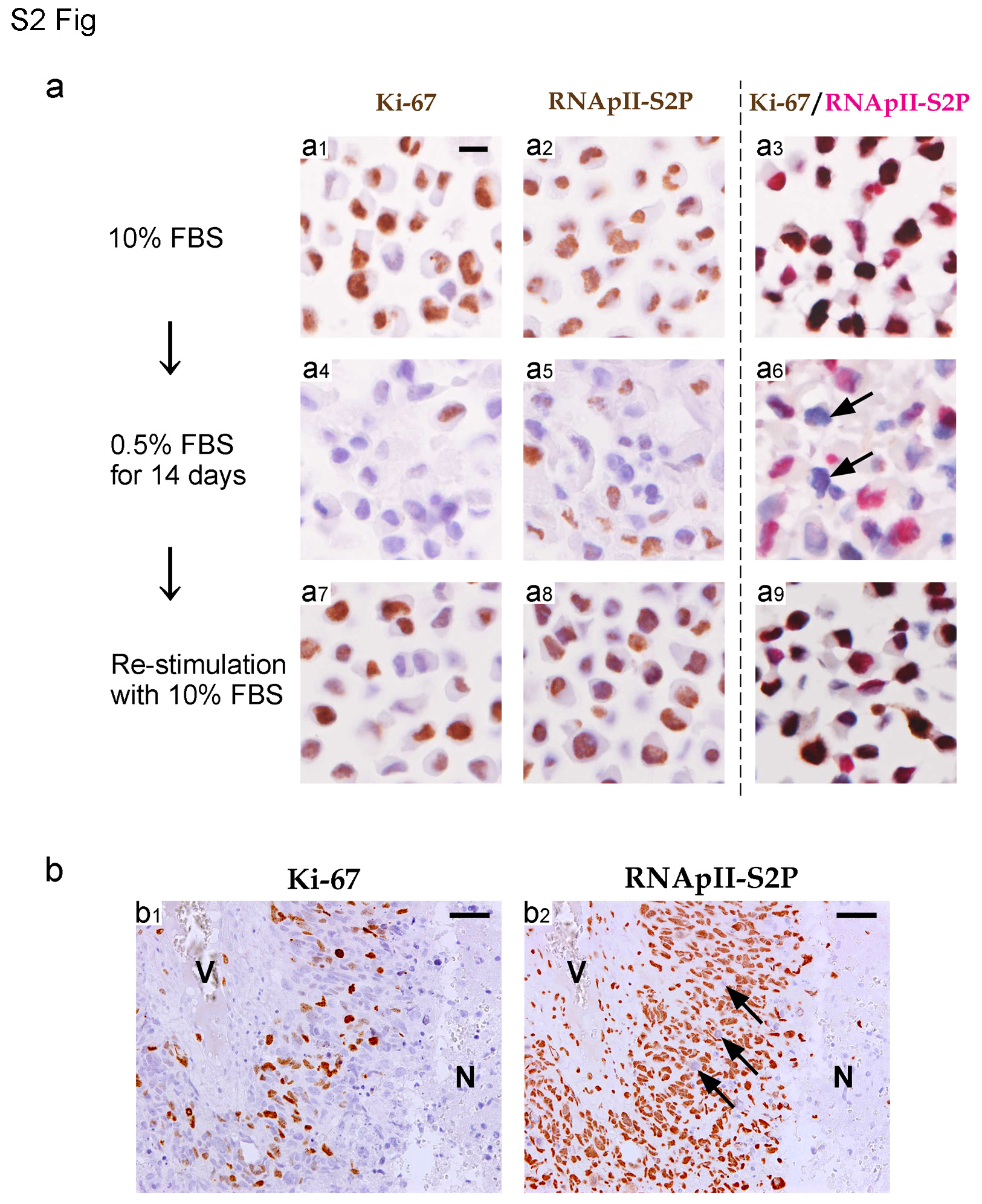

Supplement: S2 Fig — a: Regulation of Ki-67 and RNApII-S2P during proliferation and quiescence in T98G glioblastoma cells. T98G cells were grown in culture medium containing 10% (v/v) fetal bovine serum (FBS), were induced to become quiescent by serum starvation in medium supplemented with 0.5% (v/v) FBS for 14 days, and then were re-stimulated by being split 1:5 into new medium containing 10% (v/v) FBS and cultured for 3 days. The cells were detached from dishes with trypsin-EDTA solution, fixed in 10% (v/v) neutral buffered formalin, and centrifuged. Paraffin sections of the pellet were cut, and expression of Ki-67 and RNApII-S2P was examined by single (brown; a1, a2, a4, a5, a7, a8) or double immunostaining (Ki-67, brown; RNApII-S2P, red; a3, a6, a9). Hematoxylin (blue) was used as a nuclear stain. Ki-67- RNApII-S2P-/low cells (blue cells in the double stained sections) emerged only in the quiescent condition (a6, arrows). Scale bar, 10 μm. b: Single-color immunostaining for Ki-67 (b1) and RNApII-S2P (b2) in serial sections of glioblastoma tissue. Ki-67- tumor cells were frequently found, whereas only a few RNApII-S2P-/low cells (arrows) were observed around necrotic area. N, necrotic area; V, blood vessels. Scale bars, 50 μm. (JPG) [file pone.0147366.s002.jpg]

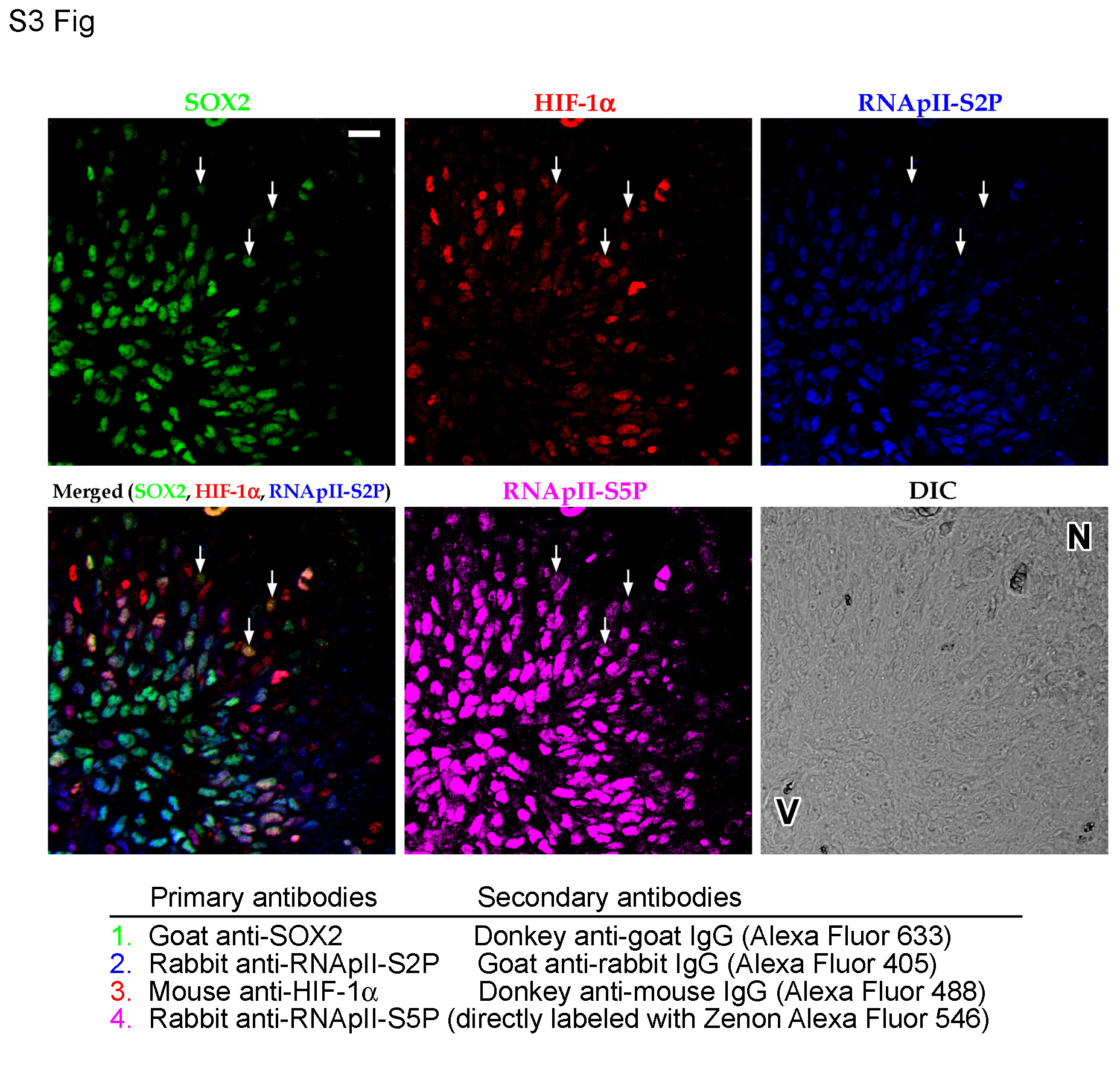

Supplement: S3 Fig — SOX2+ HIF-1α+ RNApII-S2P-/low cells (arrows) are positive for RNApII-S5P. As shown in the table (bottom), sections were incubated with goat anti-SOX2 antibody and then with Alexa Fluor 633-conjugated donkey anti-goat IgG secondary antibody. Next, rabbit anti-RNApII-S2P antibody and then Alexa Fluor 405-conjugated goat anti-rabbit IgG secondary antibody were applied. The sections were reacted with mouse anti-HIF-1α antibody and then with biotinylated donkey anti-mouse IgG secondary antibody and Alexa Fluor 488-conjugated streptavidin. Finally, rabbit anti-RNApII-S5P antibody directly labeled with Zenon Alexa Fluor 546 was applied. N, necrotic area; V, blood vessels; DIC, differential interference contrast image. Scale bar, 25 μm. (JPG) [file pone.0147366.s003.jpg]

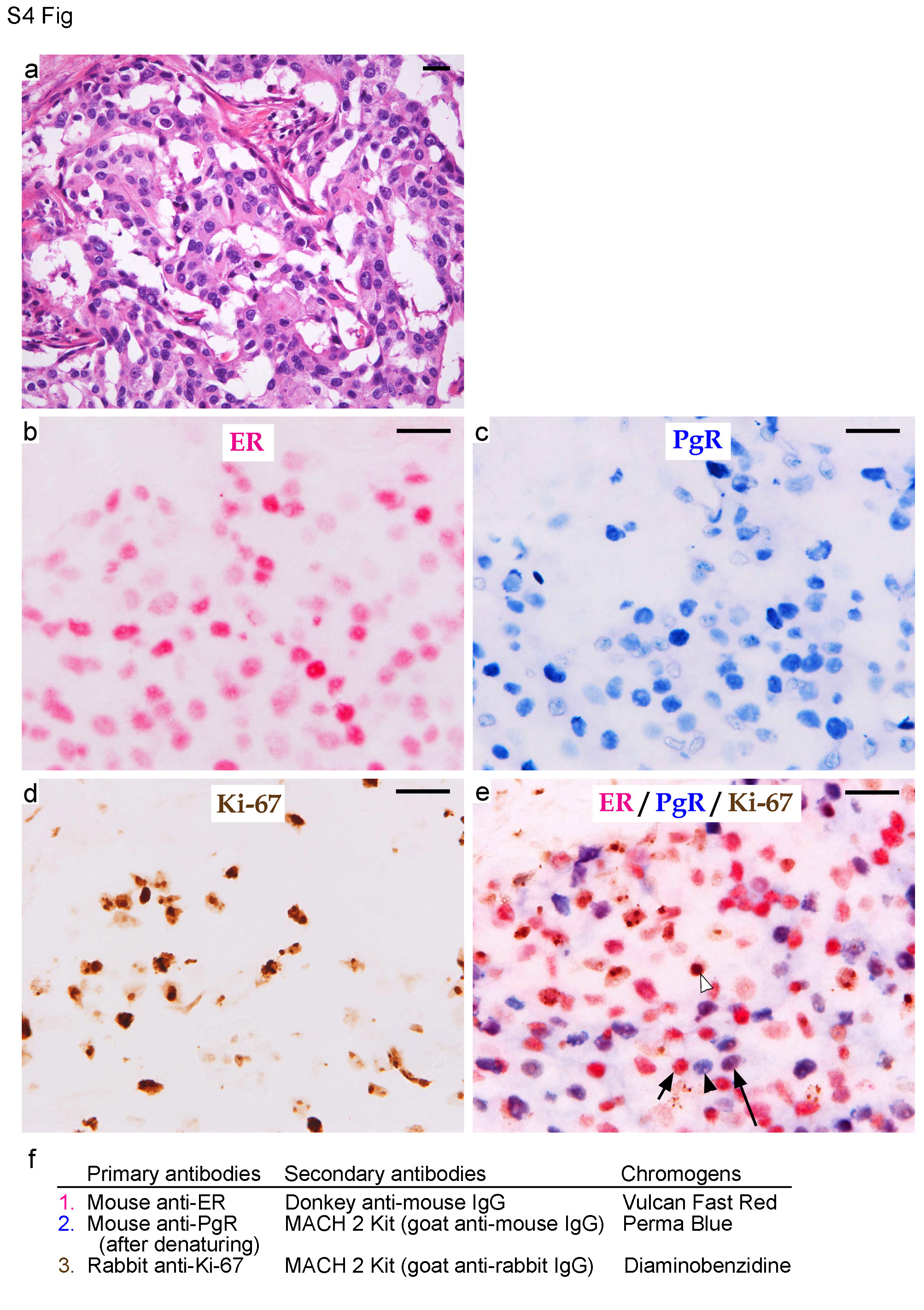

Supplement: S4 Fig — a: Hematoxylin-eosin (H-E) staining. b–e: Immunostaining of serial sections. Single immunohistochemistry for ER (b), PgR (c), and Ki-67 (d), and triple immunostaining for ER/PgR/Ki-67 (e) are shown (ER, red; PgR, blue; Ki-67, brown). In triple immunostaining (e), ER+ PgR- Ki-67- cells were stained red (short arrow), ER- PgR+ Ki-67- cells were stained blue (black arrowhead), ER+ PgR+ Ki-67- cells were stained purple (long arrow), and Ki-67+ cells were stained brown (white arrowhead). These colors are easily distinguishable. Scale bars, 25 μm. f: Summary of the staining methods used in e. Sections were incubated with mouse anti-ER antibody and then with alkaline phosphatase (AP)-conjugated donkey anti-mouse IgG secondary antibody, and color was developed with Vulcan Fast Red. After denaturing, mouse anti-PgR antibody and rabbit anti-Ki-67 antibody were applied, and then the sections were reacted with MACH 2 Double Stain 1 (a secondary antibody cocktail of AP-conjugated anti-mouse IgG and horseradish peroxidase-conjugated anti-rabbit IgG antibodies). Color was developed with Perma Blue/AP for PgR and diaminobenzidine for Ki-67. (JPG) [file pone.0147366.s004.jpg]

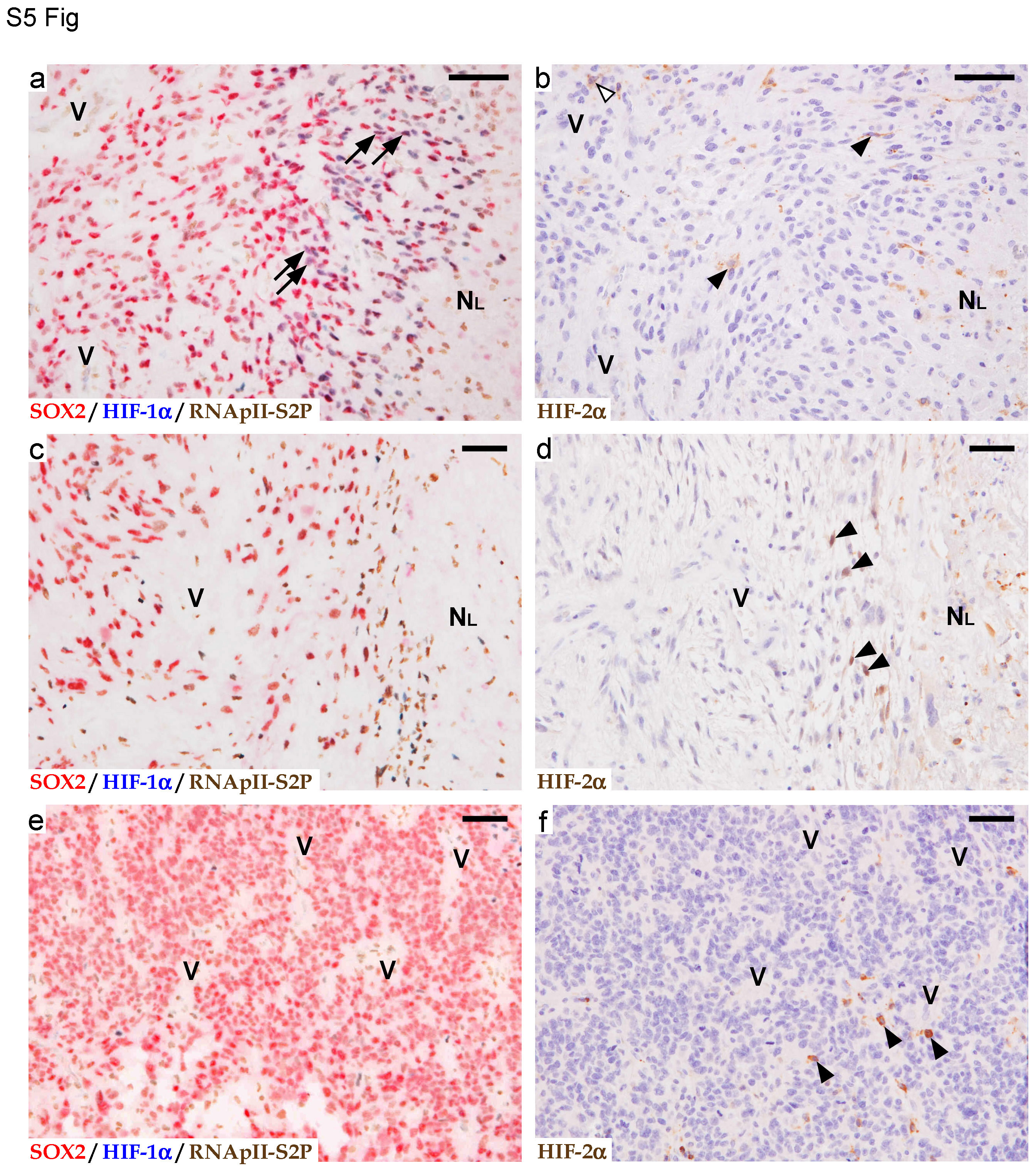

Supplement: S5 Fig — Triple immunostaining for SOX2/HIF-1α/RNApII-S2P (a, c, and e) and single immunostaining for HIF-2α (b, d, and f) are shown. a, b: Serial sections of an area around a large ischemic necrosis (NL). HIF-2α+ cells (black arrowheads in b) were found near SOX2+ HIF-1α+ RNApII-S2P-/low cells (arrows in a), but this finding was only occasionally observed. HIF-2α+ cells were also found in perivascular areas (white arrowhead in b), whereas SOX2+ HIF-1α+ RNApII-S2P-/low cells were not detected in these areas. c, d: Serial sections of another area around a large ischemic necrosis. HIF-2α+ cells were observed (arrowheads in d) whereas no SOX2+ HIF-1α+ RNApII-S2P-/low cells were found in this location (c). e, f: Serial sections of an area devoid of necrosis. HIF-2α+ cells were found near blood vessels (arrowheads in f), but no SOX2+ HIF-1α+ RNApII-S2P-/low cells were observed in this area (e). NL, large ischemic necrosis; V, blood vessels. Scale bars, 50 μm. (JPG) [file pone.0147366.s005.jpg]
